# Identifying the Characteristics of Patients With Cervical Degenerative Disease for Surgical Treatment From 17-Year Real-World Data: Retrospective Study

**DOI:** 10.2196/16076

**Published:** 2020-04-03

**Authors:** Si Zheng, Yun Xia Wu, Jia Yang Wang, Yan Li, Zhong Jun Liu, Xiao Guang Liu, Geng Ting Dang, Yu Sun, Jiao Li

**Affiliations:** 1 Institute of Medical Information & Library Chinese Academy of Medical Sciences & Peking Union Medical College Beijing China; 2 Orthopaedic Department Peking University Third Hospital Beijing China

**Keywords:** cervical degenerative disease, real-world data, inpatient surgery, mean age at surgery, sex, average length of stay

## Abstract

**Background:**

Real-world data (RWD) play important roles in evaluating treatment effectiveness in clinical research. In recent decades, with the development of more accurate diagnoses and better treatment options, inpatient surgery for cervical degenerative disease (CDD) has become increasingly more common, yet little is known about the variations in patient demographic characteristics associated with surgical treatment.

**Objective:**

This study aimed to identify the characteristics of surgical patients with CDD using RWD collected from electronic medical records.

**Methods:**

This study included 20,288 inpatient surgeries registered from January 1, 2000, to December 31, 2016, among patients aged 18 years or older, and demographic data (eg, age, sex, admission time, surgery type, treatment, discharge diagnosis, and discharge time) were collected at baseline. Regression modeling and time series analysis were conducted to analyze the trend in each variable (total number of inpatient surgeries, mean age at surgery, sex, and average length of stay). A *P* value <.01 was considered statistically significant. The RWD in this study were collected from the Orthopedic Department at Peking University Third Hospital, and the study was approved by the institutional review board.

**Results:**

Over the last 17 years, the number of inpatient surgeries increased annually by an average of 11.13%, with some fluctuations. In total, 76.4% (15,496/20,288) of the surgeries were performed in patients with CDD aged 41 to 65 years, and there was no significant change in the mean age at surgery. More male patients were observed, and the proportions of male and female patients who underwent surgery were 64.7% (13,126/20,288) and 35.3% (7162/20,288), respectively. However, interestingly, the proportion of surgeries performed among female patients showed an increasing trend (*P*<.001), leading to a narrowing sex gap. The average length of stay for surgical treatment decreased from 21 days to 6 days and showed a steady decline from 2012 onward.

**Conclusions:**

The RWD showed its capability in supporting clinical research. The mean age at surgery for CDD was consistent in the real-world population, the proportion of female patients increased, and the average length of stay decreased over time. These results may be valuable to guide resource allocation for the early prevention and diagnosis, as well as surgical treatment of CDD.

## Introduction

### Background

According to the evidence classification system for evidence-based medicine, the best evidence originates from randomized controlled trials (RCTs) and associated systematic evaluations. However, RCTs have some shortcomings, such as ethical limitations, small sample sizes, short observation times, narrow observation scopes, and high experimental costs [1]. Comparatively, real-world data (RWD), which are health care data that have been collected from different sources, including electronic health records, insurance payment and billing databases, disease registration databases, family monitoring equipment data, and mobile health devices, can be complementary sources of RCT data for establishing a more robust evidence base on the effectiveness of medicines, as well as the relative effectiveness as compared with existing products in clinical practice [2,3]. Recent studies have focused on designing and implementing evidence-based surgical safety information systems, and big data analytics on RWD can yield new and powerful insights into the effectiveness of different medicines and patient care [4-6]. RWD enable new opportunities to be explored in clinical studies. For instance, globally, various kinds of operations are performed each year, and associated surgical details recorded in electronic medical databases have great research value. Therefore, more research is needed to transform these kinds of RWD into real-world evidence, which can assist evidence-based health care decision–making systems.

In recent years, there has been growing interest in the prevention of various degenerative diseases. Essentially, all people who live a long life will develop degenerative disorders [7]. With the aging of the Chinese population, age-related degenerative disorders are becoming increasingly common and thus are worthy of attention. Cervical degenerative disease (CDD) is a consequence of aging, and it can manifest as axial neck pain, upper extremity radiculopathy, myelopathy, or some combination thereof [8-10]. The treatments for CDD vary considerably according to clinical guidelines. In most cases, nonoperative modalities are typically prescribed as the first method of choice for the conservative treatment of pain related to CDD [11-13]. Recently, the management of severely affected patients with CDD has progressed toward surgical treatments, such as anterior and posterior cervical surgeries [14,15]. Although nonoperative treatment continues to play an important role in treating these patients, surgical intervention has become the mainstay when conservative treatment fails or when patients have neurological deficits [16-18]. Nevertheless, the rate of surgical intervention, as well as the direct costs for degenerative disorders will increase with population aging, which will become a great economic burden on the health care system.

Yet, few large-scale clinical studies evaluating the demographic changes in the Chinese population receiving surgical treatments for CDD have been carried out. However, knowledge of these trends can be beneficial for medical care, surgical treatment strategy selection, and early disease prevention, and it might promote effective clinical management of this disease [19,20].

### Objectives

In light of the recently published guidelines from the US Food and Drug Administration on the communication of RWD and real-world evidence to support regulatory decision making, as well as promote evidence-based public health policies in China [21,22], we conducted a real-world study on CDD. In this study, we provide insights into the longitudinal trends and changes in the demographic characteristics of patients who received surgical treatments, which can be used to build evidence-based criteria for effective clinical management and health care system development.

## Methods

### Study Setting

**Figure 1:**
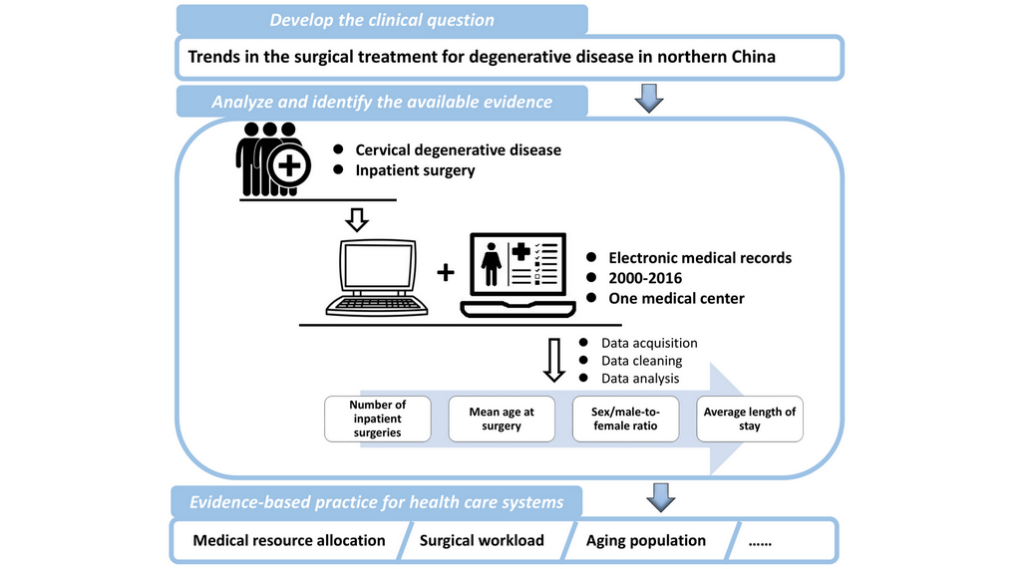
Workflow of a real-world study model.

This study was a retrospective analysis of electronic medical records (EMRs) on surgical treatments for CDD performed in the Orthopedic Department at Peking University Third Hospital (PUTH) between 2000 and 2016. The study was approved by the institutional review board (IRB00006761-M2018082). PUTH is a modernized and comprehensive upper first-class hospital that serves as a regional center for orthopedic care in northern China. Here, we designed a real-world study to analyze and assess the trends in the total number of inpatient surgeries for CDD, mean patient age at surgery, patient sex (male-to-female ratio), and average length of stay (LOS) (Figure 1).

### Patient Data Acquisition

Data on patients with CDD who were registered from January 1, 2000, to December 31, 2016, and who underwent inpatient surgical treatment in the Orthopedic Department at PUTH were collected via the health care system, and the associated data on the first page of the medical records were used. Eligible patients included Chinese patients aged ≥18 years with a principle diagnosis of CDD according to the International Classification of Diseases, 10th Revision, Clinical Modification (ICD-10-CM) code. Furthermore, inpatient surgeries were identified through ICD-9-CM procedure codes (03.02, 03.09, 14.71, 77.69, 77.79, 77.89, 77.99, 78.09, 78.59, 78.69, 80.49, 80.51, 80.99, 81.02, 81.03, 81.05, 81.08, 81.32, 81.33, 81.51, 81.62, 81.63, 81.65, 83.19, 84.51, 84.61, 84.62, and 84.66).

In this study, inpatient surgery was defined as a surgical operation or procedure involving an overnight stay in an inpatient institution. A total of 20,288 inpatient surgeries for patients with CDD met the inclusion criteria during the study period. The additional data collected included patient sex, age at surgery, admission time, discharge time, LOS, discharge diagnosis, and surgery type.

### Data Cleaning and Statistical Analysis

#### Summarization and Measures

For all analyses, we used January 1, 2000, as the start of the study period because complete data were available from this year onward. We used R version 3.6.0 (R Foundation for Statistical Computing, Vienna, Austria) for all analyses. First, patient charts were reviewed to gain insights into clinical characteristics. Analyses of the mean age at surgery and number of inpatient surgeries were stratified by sex (male and female) and age (18-30, 31-35, 36-40, 41-45, 46-50, 51-55, 56-60, 61-65, 66-70, 71-75, 76-80, and ≥81 years).

The mean age at surgery was calculated by averaging the patients’ data by either month or year. Median was used to measure the average LOS. Another key measure, the annual growth rate of the number of inpatient surgeries, was calculated as an increase in the number of operations divided by the total number of operations from the previous year.

#### Statistical Tests

We used the chi-square goodness-of-fit test to compare the number of inpatient surgeries across different groups (age and sex), and the *t*-test to compare the mean age at surgery. Additionally, the rank-sum test was used to compare the average LOS between male and female patients.

#### Time Series Analysis

Time series analysis was used to assess the variation and trends in the number of inpatient surgeries over time. We aggregated the data on patients with CDD who were enrolled from 2000 to 2016 into a monthly time series based on the admission date and analyzed the overall trend. Generally, the growth in the number of surgical operations was not stationary but instead exhibited an ascending trend and seasonal behavior. We thereafter selected the Holt-Winters exponential smoothing model for time series forecasting [23]. We specified the season length as 12 one-month periods, because we found that the number of surgeries always peaked in late spring (March) but declined in January and February. Interestingly, this pattern is likely to repeat every year.

**Figure 2:**
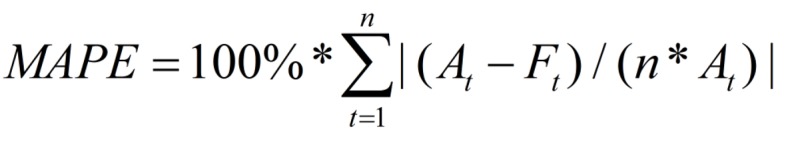
Mathematical expression of mean absolute percentage error (MAPE). A_t_: the actual value; F_t_: the forecast value.

Detailed analyses were performed according to the following steps: (1) The number of inpatient surgery admissions by month from January 1, 2000, to December 31, 2015, constituted the training set, whereas the remaining records from January 1, 2016, to December 31, 2016, constituted the testing set. Both the training set and testing set were converted into time series. (2) As the seasonal fluctuation evident in the data is not strictly distributed, both additive and multiplicative methods were applied to train the model. (3) It was assessed whether the model was sufficient. The Ljung-Box (LB) test was used to evaluate the residuals of the fitted model, and the mean absolute percentage error (MAPE) was used to evaluate the forecast error. The mathematical expression of the MAPE is shown in Figure 2.

#### Regression Analysis

A linear regression model was used to assess the variation and trends in the male-to-female ratio over the past 17 years, with demographic characteristics as dependent variables and the index calendar year as the independent variable. In this study, *P*<.01 was considered statistically significant.

## Results

### Increasing Trend in the Number of Inpatient Surgeries for Cervical Degenerative Disease

The patient demographic characteristics are shown in Table 1. In total, 20,288 inpatient surgeries for CDD were performed at PUTH over the past 17 years. Cervical disorder was the most frequent disorder within the spinal degenerative disease category [24]. Overall, differences in the number of operations for patients with CDD across age groups and sexes were statistically significant (*P*<.001; Table 1), reflecting the real-world setting of this study.

**Table 1:** Summary of the 20,288 inpatient surgery records for cervical degenerative disease and comparison of the number of inpatient surgeries across age groups and sexes.

Variable		Number of inpatient surgeries	*P* value^a^
**Sex**			<.001
	Female	7162	
	Male	13,126	
**Age at surgery, years**			<.001
	18-30	278	
	31-35	593	
	36-40	1537	
	41-45	2772	
	46-50	3524	
	51-55	3620	
	56-60	3165	
	61-65	2415	
	66-70	1496	
	71-75	690	
	76-80	172	
	≥81	26	
Total		20,288	

^a^Chi-square goodness-of-fit test.

**Figure 3:**
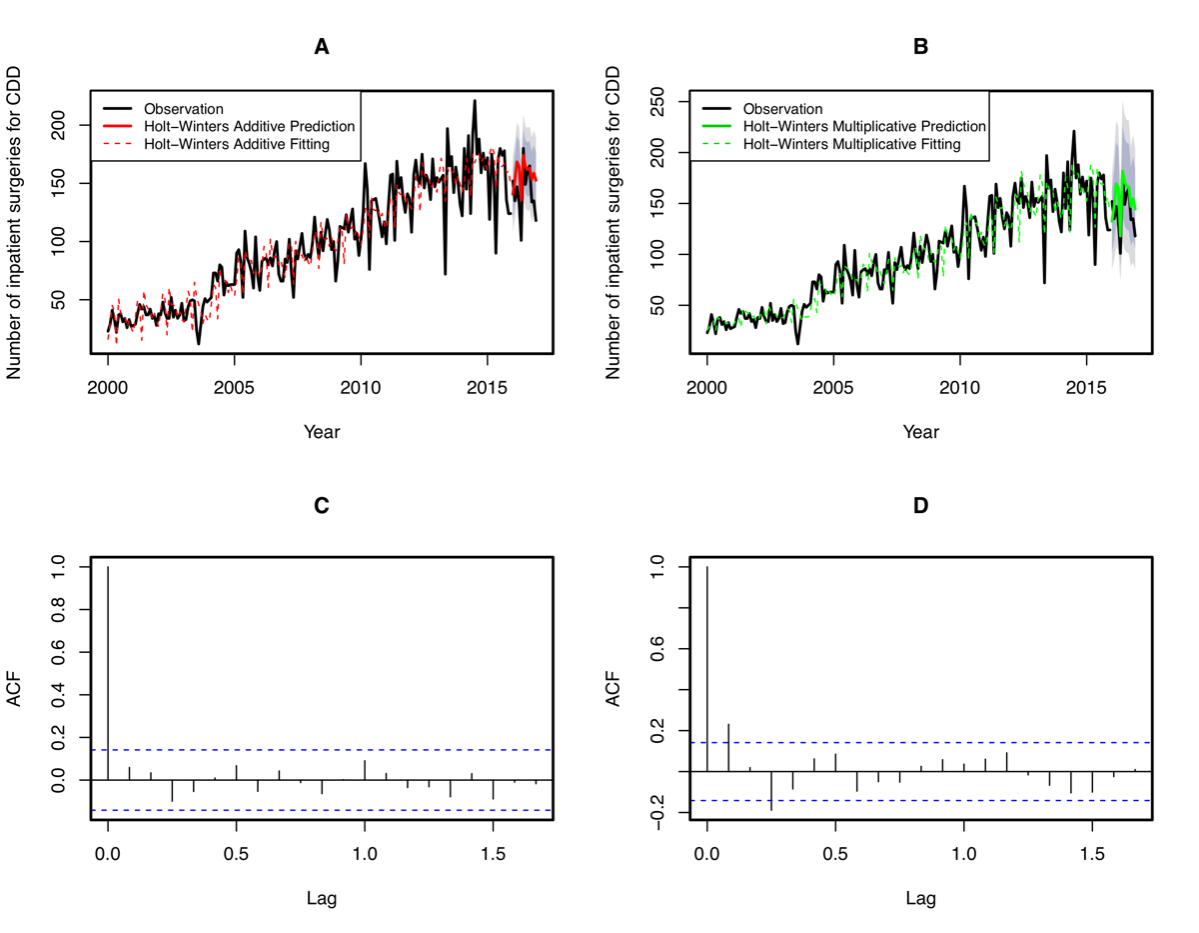
Observed, fitted, and predicted numbers of inpatient surgeries for cervical degenerative disease (CDD). Time series analyses using Holt-Winters additive and multiplicative method were conducted to compare the observed and predicted numbers of surgeries from January 2000 to December 2016. (A) The black solid line represents the observed number of surgeries. The red dotted line represents the fitted number of surgeries determined with the additive method. The red solid line represents the predicted number of surgeries determined with the additive method using January 2000 to December 2015 as an observation base. The 80% CIs and 95% CIs are denoted by dark gray and light gray areas, respectively. (B) The black solid line represents the observed number of surgeries. The green dotted line represents the fitted number of surgeries determined with the multiplicative method. The green solid line represents the predicted number of surgeries determined with the multiplicative method using January 2000 to December 2015 as an observation base. The 80% CIs and 95% CIs are denoted by dark gray and light gray areas, respectively. (C) Autocorrelation function (ACF) plot for the time series model generated with the Holt-Winters additive method. (D) ACF plot for the time series model generated with the Holt-Winters multiplicative method.

From 2000 to 2016, there was a significant increase in the overall number of CDD surgery cases; it increased from 374 in 2000 to 1709 in 2016, with an average annual increase (growth rate) of 11.13%. The number of inpatient surgeries fluctuated seasonally; it declined in January and February but always peaked in late spring, and the fastest monthly growth rate was always noted in March. Time series analysis was adopted to identify the underlying structure and function (number of inpatient surgeries [monthly]; additive method: LB test *P*=.92, MAPE=16.44%; multiplicative method: LB test *P*=.03, MAPE=14.37%), and both methods were capable of predicting the seasonal peak in most months (Figure 3). Comparatively, the multiplicative method had a relatively higher forecasting accuracy. The MAPEs of the forecasting error in the testing set for the additive and multiplicative methods were 14.18% and 12.13%, respectively.

### Consistency in the Mean Age at Surgery for Cervical Degenerative Disease

Overall, 88.1% (17,880/20,288) of the surgeries for CDD were performed in patients older than 40 years. The number of CDD surgeries differed by age group and was especially high in patients aged approximately 50 years. Specifically, 76.4% (15,496/20,288) of the surgeries were performed in patients aged 41-65 years and 50.8% (10,309/20,288) were performed in those aged 46-60 years. The average age at the time of inpatient surgery was 52.58 and 52.92 years among male and female patients, respectively.

**Figure 4:**
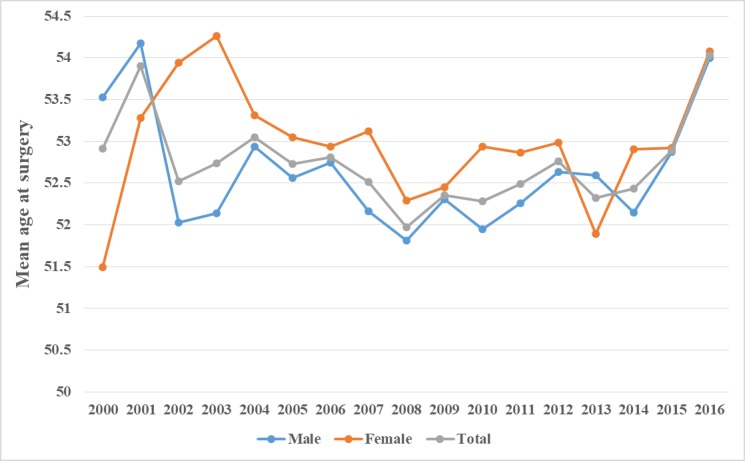
Trends in the mean age at surgery for cervical degenerative disease by sex (2000-2016).

**Figure 5:**
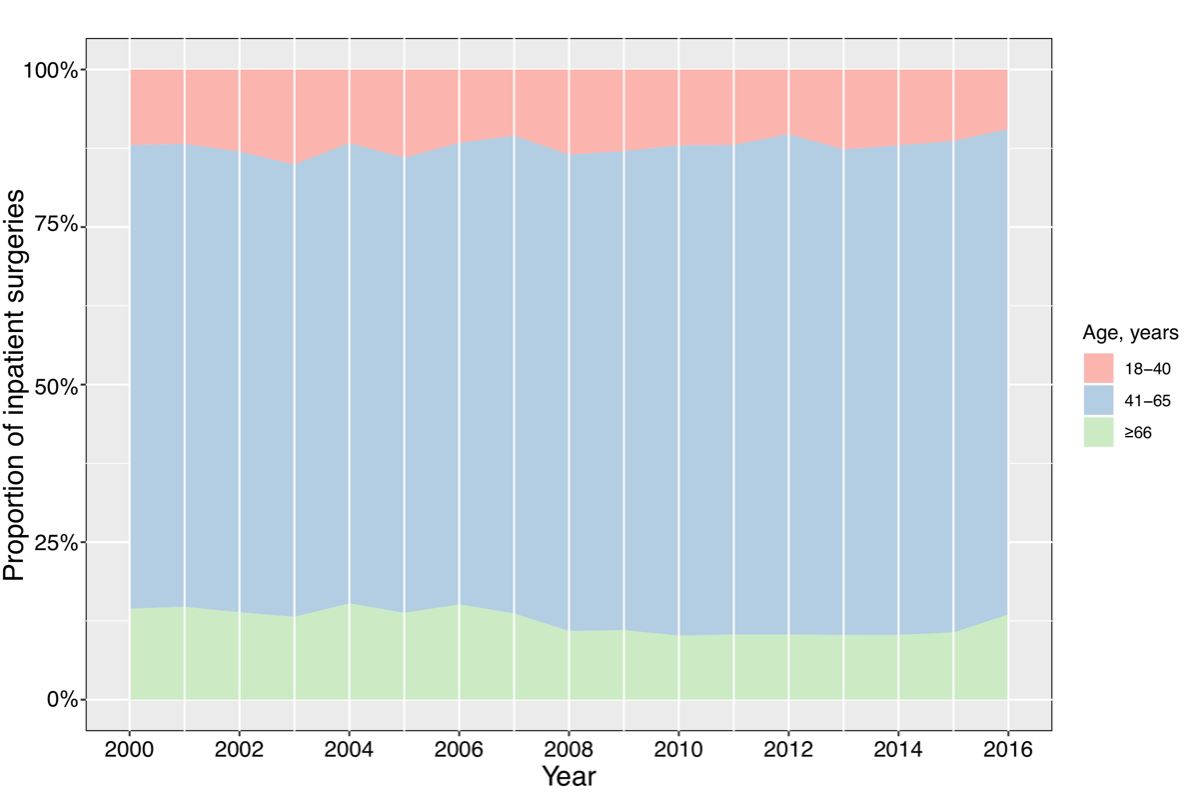
Annual proportion of inpatient surgeries for cervical degenerative disease among patients in different age groups (2000-2016).

Over the past 17 years, there was no statistically significant change in the mean age at surgery (yearly) among the patients with CDD (coefficient of variation=0.01) (Figure 4). In our study population, the number of surgical treatments did not show a trend toward younger patients in recent years. Moreover, there was no significant difference in the mean age at surgery (yearly) between male and female patients (*t*-test, *P*=.16). Overall, the mean age at surgery for patients with CDD remained consistent, except for a sudden increase in 2016. The overall proportion of surgeries performed in the elderly population (older than 70 years) over the past 17 years was very small (yearly, 2.94%-5.52%); thus, the mean age at CDD surgery was not affected by population aging (life expectancy at birth for the Chinese population has increased by more than 4.94 years since 2000; it was 71.4 years in 2000 and 76.34 years in 2015) [25]. Upon further analysis of the population changes in the different age groups, we found that the proportions of patients aged 41 to 65 and ≥66 years in the overall population structure showed a relatively small increasing trend during the study period (with an average annual growth rate of 0.30% and 0.14%, respectively) (Figure 5). In comparison, the proportion of patients aged 18 to 40 years decreased slightly. However, these small variations in the patient population structure had no significant effect on the mean age at surgery for CDD.

### Decrease in the Male-to-Female Ratio Among Patients With Cervical Degenerative Disease

Both the number of inpatient surgeries performed in male patients and the number of inpatient surgeries performed in female patients showed an increasing trend (Table 2). Interestingly, the male-to-female ratio among patients who received surgical treatment was 1.83:1 (13,126 male and 7162 female patients, respectively). Based on the annual statistics from the National Bureau of Statistics of China, the male-to-female ratio in the Chinese population was only approximately 1.05 to 1.07 during the study period [25]. Specifically, the number of inpatient surgeries was higher in male patients than in female patients. In fact, when “working hours” is considered as only the time spent at the office, many male individuals work more hours as compared with female individuals in East Asian countries, and the income of female individuals is lower than that of male individuals [26]. More attention, including more effective allocation of health care resources, should be paid to the male population, especially those in the older age group.

**Table 2:** Annual number of inpatient surgeries performed among patients with cervical degenerative disease grouped by sex (2000-2016).

Year	Surgeries in male patients, n	Surgeries in female patients, n	M/F^a^	M/F_C^b,c^
2000	260	114	2.28	1.07
2001	307	134	2.29	1.06
2002	352	124	2.84	1.06
2003	333	131	2.54	1.06
2004	560	219	2.56	1.06
2005	632	333	1.90	1.06
2006	635	318	2.00	1.06
2007	657	381	1.72	1.06
2008	819	402	2.04	1.06
2009	824	435	1.89	1.06
2010	984	502	1.96	1.05
2011	1044	610	1.71	1.05
2012	1166	655	1.78	1.05
2013	1099	714	1.54	1.05
2014	1272	763	1.67	1.05
2015	1144	656	1.74	1.05
2016	1038	671	1.55	1.05

^a^Ratio of the number of inpatient surgeries performed in male patients to the number of inpatient surgeries performed in female patients.

^b^Male-to-female ratio in the Chinese population.

^c^Calculation of M/F_C was based on data from the National Bureau of Statistics of China [25].

**Figure 6:**
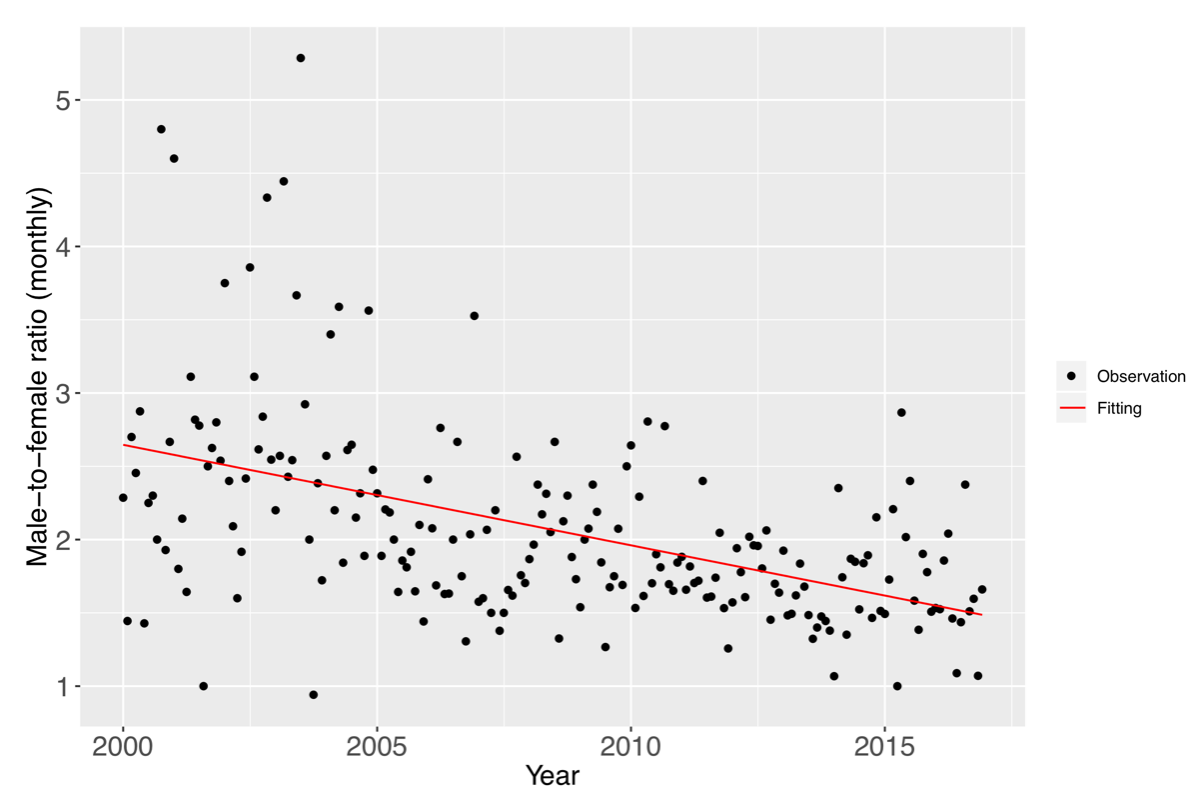
Trends in the number of inpatient surgeries performed among male and female patients. The proportion of surgeries performed among female patients increased in the past 17 years (male-to-female ratio [monthly], linear regression *P*<.001), and the line of best fit is plotted in red.

On the other hand, interestingly, a narrowing trend in the sex gap was observed. We found that the male-to-female ratio among the patients who underwent surgery for CDD showed a decreasing trend (male-to-female ratio [monthly], linear regression: *P*<.001) (Figure 6). Female patients with CDD progressed to surgery at a faster rate as compared with male patients in the past 17 years, particularly since 2008. This result may be due to an increase in the female employment rate; improper sitting habits; and occupational hazards, such as the transportation of goods by bearing weight on the top of the head, which might increase the risk for CDD [27]. In addition, although female individuals might leave the labor force upon marriage or childbirth, they re-enter when they are middle-aged, and some female individuals may remain in the labor force after marriage and childbirth.

### Decreasing Tendency in the Average Length of Stay

The LOS for the surgical treatment of CDD decreased by 15 days over the last 17 years (decreased from an average of 21 days in 2000 to 6 days in 2016, and the average rate of decrease was 6.87%), and the largest decreases were noted from 2000 to 2001 and from 2006 to 2007 (Table 3). This result may be due to a better understanding of disease pathogenesis, recent advances in diagnosis and operative techniques, and improvements in standard hospital ward management [28,29]. There was no significant difference in the average LOS (yearly) between male and female patients (Wilcoxon rank-sum test, *P*=.64). As the LOS in our study had a slight right-skewed distribution, we used the median to measure the average LOS.

**Table 3:** Average length of stay for cervical degenerative disease inpatient surgeries categorized by sex (2000-2016).

Year	Average LOS^a^ among male patients, days	Average LOS among female patients, days	Average LOS among all patients, days
2000	22	21	21
2001	18	20	19
2002	19	18.5	19
2003	17	18	17
2004	16	16	16
2005	14	15	14
2006	13	13	13
2007	10	10	10
2008	9	9	9
2009	8	8	8
2010	7	7	7
2011	7	6	6
2012	6	5	5
2013	6	5	5
2014	6	5	6
2015	6	5	5
2016	6	5	6

^a^LOS: length of stay.

**Figure 7:**
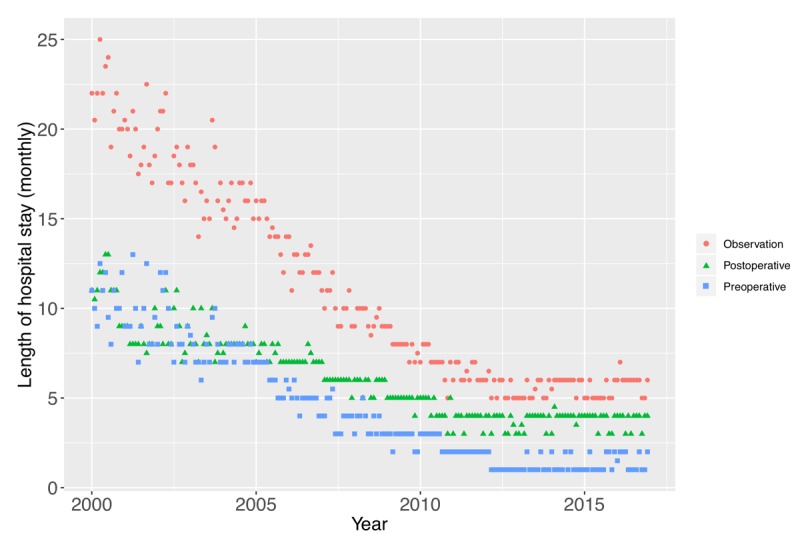
Trends in the average length of stay (LOS) for cervical degenerative disease surgical treatment. The total LOS is plotted with red dots (average LOS [monthly]), postoperative LOS is plotted with green triangles, and preoperative LOS is plotted with blue squares.

We further observed an overall decrease in the LOS, and this trend might continue in subsequent years, but at a slower rate (Figure 7). Moreover, the preoperative LOS decreased at a faster rate as compared with the postoperative LOS (Figure 7). There is evidence that the use of ambulatory surgery (1-day hospital stay), which represents a more comfortable and less expensive alternative to conventional surgery, has already been established in many hospitals in the United States and Europe, because it can minimize the impact of hospitalization and promote the early recovery of patients [30,31]. Our data demonstrate that such a trend might develop substantially in China in the future.

## Discussion

### Principal Findings

In this real-world study, we assessed data on surgical treatments performed in patients with CDD at a hospital located in northern China from 2000 to 2016. The trends can be characterized by an increase in the number of inpatient surgeries performed for CDD over the past 17 years in PUTH, a consistent mean age at surgery, and a decreased average LOS for surgical treatment. In addition, inpatient surgeries performed among female patients accounted for an increasing proportion of the total number of procedures. Our study is one of the first retrospective studies to analyze the surgical treatment of CDD in a large Chinese population. Some of these findings were, in general, similar to those of previous studies that focused on other degenerative disorders in other countries [19,32,33].

The rapid development of information technologies, such as EMRs used in medical care systems, allows the timely and secure exchange of health information across physicians, hospitals, specialists, patients, and health care insurers; therefore, information technologies can significantly assist providers in obtaining meaningful information. The utilization of RWD retrieved from EMRs provides abundant opportunities for information-based improvements in designing and conducting clinical trials and studies in the health care setting to answer questions that were previously thought to be unanswerable. Previous studies have shown that real-world evidence derived from sources outside typical clinical research settings, such as electronic health records, can help with patient care, research on health care systems, and quality improvement [1,34]. An example of extracting knowledge from multisourced clinical data to support clinical decisions is the development of an evidence-based stratified surgical safety information system based on the formulated framework [5]. Other studies have developed evidence-based educational tools to assist clinicians in making clinical decisions for patients with degenerative diseases in North America [35,36].

In this study, we focused on the use of EMRs for identifying trends in factors related to the surgical treatment of CDD. First, as we discussed above, surgical treatment was focused primarily on individuals aged 41 to 65 years, as the mean age at the time of inpatient surgery was 52.58 years for male patients and 52.92 years for female patients. A study in Norway showed that the average age difference was 1.4 years, as the mean age at the time of surgery was 50.8 years for female patients and 52.2 years for male patients [37]. In the United States, the mean age of patients has increased [38-40], whereas in northern China, no relevant change in the mean age at surgery for CDD has occurred. A similar study was conducted in Finland [41], in which the mean age of patients actually increased by fewer years as compared with that in the catchment population. A previous study reported that the prevalence of cervical spondylosis was approximately 30% in younger age groups [24]. In this study, we found that the proportion of surgical operations for CDD among individuals aged 18 to 30 years was approximately 1.4% (278/20,288) and that the highest proportion of operations was among individuals aged 41 to 65 years but not among older individuals. Thus, the severe form of CDD that does not respond well to nonsurgical forms of treatment and requires inpatient surgery is relatively more common in middle-aged and aging adults, but the prevalence of this form of the disease or the necessity of surgery might decrease once individuals reach a certain age (ie, 70 years). On the other hand, younger people also have cervical disorders [26], but they appear to be managed nonsurgically. The factors discussed above might explain why the mean age at surgery has remained consistent in the period of this study. With population aging [42], initiatives that promote the clinical practice of health management for CDD in China should focus on middle- and old-age groups (age groups of 41-65 years, etc) that have constituted the primary patient population in the past 12 years. For instance, to raise public awareness about CDD and help physicians identify, diagnose, and manage this kind of degenerative disease more effectively, radiological evaluation of the cervical area should be included in the annual health examination and efficient CDD screening programs, such as magnetic resonance imaging programs, should be promoted for male and female individuals aged 41 to 65 years [43,44]. In addition, the hospital should incorporate the opinions of experts in the fields of spine surgery, neurology, rehabilitation medicine, and physiatry to help identify additional clinical and imaging predictors of the diagnoses and surgical outcomes in aging populations and determine which patients are most likely to benefit from surgical intervention.

Second, the proportion of surgeries performed among female patients showed an increasing trend over the past 17 years in this study; more female patients than male patients with CDD were admitted for inpatient surgery, and the narrowing gap between the sexes was similar to the results reported in our previous study [45]. Nevertheless, similar to the observations in other studies on cervical diseases, more surgical operations were performed among male patients than among female patients [46-48]. There are multiple reasons for this occurrence. Although the main pathogenesis of CDD is aging, it is not the only cause; other factors, such as the effects of heavy or sedentary work, can also be major contributors [27]. Generally, this relation might reflect the fact that the rate of labor force participation related to CDD among male individuals is higher than that among female individuals in China, but the sex gap in employment has narrowed over the past decades. These results may serve to guide policy decisions pertaining to resource allocation. For instance, a reduction in the waiting time for the diagnosis and treatment of female patients and financing of the increasing surgical treatment costs for female patients by expanding health insurance coverage.

Third, we also observed that the CDD patient group that received surgical treatment had a decreasing average LOS. This decreasing trend in the length of hospital stay following spine procedures was also reported in other studies [49], and this trend might signify the standardization of postoperative protocols and implementation of effective strategies for patient surgical preparation. Thus, improvements in the decision-making process for surgical strategies [50,51], medical techniques such as imaging techniques, and hospital management, as well as the growing number of both surgeons and clinical assessments (related to increases in the numbers of nurses and technicians) in PUTH might have contributed to the shortened LOS [52]. Additionally, where possible, hospitals have incrementally added more beds by optimizing space and converting administrative areas into medical facilities and bed space. Similar strategies for converting existing space from less to more needed services should be established and encouraged. In the future, LOS will likely decrease, and facilities will be transformed into ambulatory surgery centers with shorter waiting times, tighter scheduling control, more specialized surgical teams, and faster turnaround times [30,53]. All these measures should be encouraged, as they can help in making allocation decisions that minimize the amount of resources wasted in ineffective or inappropriate operative treatments.

Finally, a growing rate of surgery for CDD was observed in northern China over the past decade. In the context of an aging population, the prevalence of spine surgery will continue to increase owing to the progressive nature of CDD [37,41,54]. A study in the southeast United States reported that the highest annual incidence of cervical disc herniation between 1976 and 1990 was among individuals in their 60s [55]. However, in many cases, it is unclear whether CDD conditions should be treated surgically or conservatively. The technological advances in surgery and anesthesiology make operative treatment safe and more accessible [39,41]. Currently, the application of excellent imaging modalities, such as magnetic resonance imaging, enables the evaluation of degenerative diseases with high sensitivity and specificity [56]. In fact, factors other than aging, including public health awareness and nonsurgical advancements, such as health insurance policies and the establishment of a home care–dominated geriatric care system, can also influence the prevalence and treatment of the disease [57,58]. China has conducted a series of health reforms over the past two decades, and health insurance coverage is nearly universal among middle-aged and older Chinese people [59]. These factors can lead to an increase in the number of inpatients. In light of this situation, the Chinese government has focused on a prevention-oriented strategy, early diagnosis and treatment, and the promotion of the concept of a healthy lifestyle, all of which can help to reduce the rate of surgical treatment for CDD [60,61]. On the other hand, hospitals, as public health service organizations, should popularize CDD prevention knowledge and increase awareness regarding CDD in the entire population. Although surgery for CDD is associated with great and clinically important improvements in quality of life, the incremental cost-utility estimates should be well controlled within generally accepted thresholds [62].

### Limitations

There were several limitations in this study. This was a real-world study investigating the surgical treatment of patients with CDD, who were admitted to one hospital and who underwent surgery at that hospital, and the single-center nature of this study may limit external validity. In addition, we were unable to measure several important outcomes following surgical treatment. For instance, the comorbidities of CDD in the aging population, as well as comparisons between different surgery types were investigated to a limited extent. These factors might affect our results, and we will analyze these factors in our future work. Additionally, we found that few patients with CDD underwent several inpatient surgeries. Moreover, there were sudden decreases in the monthly number of inpatient surgeries owing to fortuitous events, such as medical insurance settlement and celebration activities, and no details on these factors were provided in this research. Despite these limitations, our study presents certain variations and real-world trends in Chinese patients who underwent cervical surgery and addresses the potential factors that may have influenced inpatient surgery, which will have important implications in advancing health care resource allocation methods used in medical decision making. Unfortunately, assessment of benefits is not as straightforward as the term might suggest, and the line among effective, ineffective, and experimental treatments is often a personal decision made by an individual clinician. By assessing EMR data to estimate the trends in medical treatments for CDD, we can develop effective resource allocation strategies to maximize the benefits in the population.

### Conclusions

Through a large-scale real-world population study on surgical treatments for CDD in a hospital in northern China, we provide real-world evidence that CDD may increase the workload for hospitals in China. An increased number of inpatient surgeries was found, suggesting an increasing demand for specialists and medical assistants in the surgical management of this disease. We suggest that more attention should be given to the aging population, as well as the middle-aged female population. Additionally, more discussions and heightened awareness of cervical/skeletal health are needed. The decrease in LOS suggests improvements in surgical techniques and health care systems; however, more attention should be paid to surgical care and follow-up.

## Abbreviations

CDD: cervical degenerative disease
CM: Clinical Modification
EMR: electronic medical record
ICD: International Classification of Diseases
LB: Ljung-Box
LOS: length of stay
MAPE: mean absolute percentage error
PUTH: Peking University Third Hospital
RCT: randomized controlled trial
RWD: real-world data
